# Medical News and Miscellany

**Published:** 1897-04

**Authors:** 


### Medical News and Miscellany.

**Dr. J. Frank Thornton** has removed from Columbus to Plum, Texas.

**Dr. H. Whitfield Cummings,** of Hearne, has returned from New York where he has been attending a course at the polyclinic.

**Dr. J. S. Watson** has removed from Burnet to Manor, Texas. Dr. Watson, the Manor people will find a most worthy successor of the lamented Gregg.

**Dr. S. E. Milliken,** late of New York, well known to the Texas profession, has settled in Dallas, Texas, and formed a copartnership with Dr. C. M. Rosser.

**Cards** are out announcing the approaching marriage of our handsome young friend, Dr. Joe S. Wooten, of Austin, to Miss Blossom Greenwood, of Palestine, Texas, April 21st.

**Hymeneal.**—The JOURNAL acknowledges the receipt of cards announcing the marriage, to take place at Fort Worth, April 16th (inst.), of Dr. W. A. Duringer to Miss Bernice Juaniata Hovey, all of that city.

**Any pharmacist** desiring the services of the right kind of a young man for the prescription and pharmacy business, will do well to write to J. B. C., care of this JOURNAL. See ad. The editor endorses the advertiser.

**Miss Florence A. Blunt,** daughter of ex-quarantine officer W. F. Blunt, late of Galveston, now of Lockhart, was married on the 6th of April (inst.) to Mr. Alva P. Jordan, a prominent young railroad man well known throughout the State.

**Distinguished Guests.**—The JOURNAL learns that Prof. J. W. Cale, M. D., of the Woman's Hospital, St. Louis, and Prof. E. Lanphear, M. D., editor of the *American Journal of Surgery and Gynecology*, St. Louis, will attend the Paris meeting of our State Medical Association on the 27th of this month. They will each read a paper. Prof. Lanphear has recently been the recipient of the degree LL. D.

**Bear in mind** that the meeting of the Texas State Medical Association will take place at Paris, Texas, on the fourth Tuesday of this month—April, which comes on the 27th instead of 22nd, as announced by President Loggins, in our last issue.

---

**A graduate** of the School of Pharmacy of the Medical Department of the University of Texas wishes a place as prescription clerk; has experience in the work, and can furnish testimonials of his skill. Address J. B. C., Austin, Texas, care of TEXAS MEDICAL JOURNAL.

---

**Special Rates to the Meeting of the Texas State Medical Association,** Paris, Texas, April 27th to 30th, have been made by the Gulf, Colorado and Santa Fe Railway. The rate will be one and one-third fare on the certificate plan. Ask your nearest G., C. & S. F. ticket agent, or write to W. S. Keenan, general passenger agent, Galveston, Texas, for full particulars. This road is one of the best equipped in the State, and you are assured of every comfort and the most courteous attention.

---

**The memorial** of the State Medical Association to transfer the State quarantine to the Federal government was presented to the senate and referred to the committee on federal relations on the 22nd of March. The committee returned it to the senate with the report that the quarantine laws of the State need no amendment or change.

At this writing the memorial has not been introduced in the House. The Association's committee were not present. The State Health Officer, we learn, was present by invitation, but made no remarks.

---

**Our Dead.**—It is highly probable that members of the State Medical Association have died since last meeting, of which fact the committee on Necrology have not heard. The undersigned will esteem it a courtesy if the brethren will report to either of us the death and date of death of any of their confreres, together with such data as may be in their possession, in order that proper record may be made of it in the archives of the Association, and the usual tribute be paid to their memory.

F. E. DANIEL, M. D., Chairman,
Austin, Texas.
B. F. BRITTAIN, M. D., Secretary,
Arlington, Texas.

**Mississippi.**—Our friend and quandam contemporary of the *Mississippi Medical Monthly*, which suspended some time ago, announces that, beginning with April (inst.), he will begin the publication of the *Medical Record of Mississippi*, at Biloxi, Miss. Dr. Haralson is the State Quarantine Officer at Biloxi, his home being at Forest, Miss. We are glad to know that our old State is to again have a medical journal, and we know of no one more competent to conduct a creditable journal than friend Haralson. Success to the venture!

---

**Meeting of American Medical Publishers' Assscia= tion.**—The Fourth Annual Meeting of the American Medical Publishers' Association will be held in Philadelphia on Monday, May 31st, 1897 (the day preceding the meeting of the American Medical Association). Editors and publishers, as well as every one interested in medical journalism, are cordially invited to attend and participate in the deliberations. Several very excellent papers are already assured, but more are desired. In order to secure a place on the program, contributors should send titles of their papers at once to the secretary, Chas. Wood Fassett, St. Joseph, Mo.

---

**The Texas Eye, Ear and Throat Charity Hospital** at Austin. We have received the first annual report of this institution. It makes a most gratifying showing, and there is no doubt it will become in time a State institution. It was originated and put into operation by Dr. H. L. Hilgartner assisted by the ladies of Austin, and it has been maintained, for the most part, by donations. The report shows, however, that it is already, nearly self-supporting, the receipts from pay-patients having been the first year $2550, as against a total expenditure of $4075.70 (including equipments).

Dr. Langhorn and J. N. Chadwick, of Chappel Hill, Texas, have sent Dr. Hilgartner, each, $100 as a donation to the good cause; an example worthy of imitation.

---

**A Straw.**—The Texas legislative committee on federal relations has recommended that no changes be made in the present quarantine laws of the State. This action was taken on a petition from the Texas State Medical Association, asking that the coast and border quarantine of Texas be taken from the control of the State and placed under federal control. The *Review* is indeed glad to see that there are yet men in Texas who have confidence in her ability to manage her own affairs, and who are sufficiently imbued with good old democratic doctrines to be opposed to the spirit of centralization that seems abroad in the land.—*Johnson County Review*, *Cleburne*, *Texas*.

**It is with** some satisfaction that the JOURNAL presents herewith the announcement and full program for the Paris meeting. In order to do so we held back one form till the 7th inst., our publication day being the 1st, as well as to oblige our courteous Secretary, who wrote us that the delay was caused especially by the chairmen of certain sections, and that the fault was not his. Chairmen should, by now, be impressed with the necessity of promptly sending in their reports to the secretary, else he is cramped for time, and often has to issue an incomplete program. Members should preserve this copy of the JOURNAL, as there will be but one issue of the program.

